# A Retrospective Multi-Institutional Cohort Analysis of Clinical Characteristics and Outcomes in Dedifferentiated Chondrosarcoma

**DOI:** 10.3390/cancers15092617

**Published:** 2023-05-05

**Authors:** Nam Bui, Hilary Dietz, Sheima Farag, Angela C. Hirbe, Michael J. Wagner, Brian A. Van Tine, Kristen Ganjoo, Robin L. Jones, Vicki L. Keedy, Elizabeth J. Davis

**Affiliations:** 1 Stanford Cancer Institute, Palo Alto, CA 94304, USA; 2 Vanderbilt University Medical Center, Nashville, TN 37232, USA; 3 Institute of Cancer Research, Royal Marsden Hospital, London SW3 6JJ, UK; 4 Siteman Cancer Center, Washington University in St. Louis, St. Louis, MO 63110, USA; 5 Fred Hutchinson Cancer Center, University of Washington, Seattle, WA 98109, USA

**Keywords:** sarcoma, bone sarcoma, chondrosarcoma, dedifferentiated chondrosarcoma

## Abstract

**Simple Summary:**

Dedifferentiated chondrosarcoma (DDCS) is a rare cancer that is aggressive and leads to high patient mortality. There are limited data on the efficacy of systemic treatments and the optimal agents to use. In this study, we pooled patient outcomes from five large academic sarcoma referral centers and analyzed patient characteristics, treatments received including surgery, radiation, and/or chemotherapy, as well as patient outcomes. We found that in general, prognosis was poor, with the long-term survival being only 32%, in patients diagnosed with localized cancer. Chemotherapy was the most widely used systemic therapy, but even with its use, patients had poor outcomes with a low rate of tumor shrinkage (4.9%). A few patients had prolonged tumor stability upon treatment with newer agents, such as VEGF inhibitors (pazopanib) and immune checkpoint inhibitors. Future studies should focus on exploring new therapeutics for this devastating disease.

**Abstract:**

Background: Dedifferentiated chondrosarcoma (DDCS) is a rare subset of chondrosarcoma. It is an aggressive neoplasm characterized by a high rate of recurrent and metastatic disease with overall poor outcomes. Systemic therapy is often used to treat DDCS; however, the optimal regimen and timing are not well defined, with current guidelines recommending following osteosarcoma protocols. Methods: We conducted a multi-institutional retrospective analysis of clinical characteristics and outcomes of patients with DDCS. Between 1 January 2004 and 1 January 2022, the databases from five academic sarcoma centers were reviewed. Patient and tumor factors, including age, sex, tumor size, site, location, the treatments rendered, and survival outcomes, were collected. Results: Seventy-four patients were identified and included in the analysis. Most patients presented with localized disease. Surgical resection was the mainstay of therapy. Chemotherapy was used predominantly in the metastatic setting. Partial responses were low (n = 4; 9%) and occurred upon treatment with doxorubicin with cisplatin or ifosfamide and single-agent pembrolizumab. For all other regimens, stable disease was the best response. Prolonged stable disease occurred with the use of pazopanib and immune checkpoint inhibitors. Conclusions: DDCS has poor outcomes and conventional chemotherapy has limited benefit. Future studies should focus on defining the possible role of molecularly targeted therapies and immunotherapy in the treatment of DDCS.

## 1. Introduction

Chondrosarcomas are a heterogeneous group of cartilaginous tumors that account for 20% of primary bone neoplasms [1,2]. Dedifferentiated chondrosarcoma (DDCS) is a rare, aggressive subset that accounts for 9–11% of all chondrosarcomas [3]. This histology was first described in 1971 by Dahlin and Beabout as a tumor with an area of low-grade chondrosarcoma juxtaposed with another high-grade, non-cartilaginous sarcoma, such as fibrosarcoma, undifferentiated pleomorphic sarcoma, or osteosarcoma [4]. DDCS typically affects patients in their mid-60s and has a slight predilection for males [5].

Surgery is the mainstay of therapy for localized DDCS and can be curative in a subset of cases. Radiation is generally of limited utility but can offer benefit in the metastatic setting for palliation of symptoms. Systemic therapy can be considered in both the neo/adjuvant and metastatic settings. Both NCCN and ESMO guidelines recommend treatment of DDCS with osteosarcoma-type chemotherapy [6,7]. Therefore, regimens commonly used to treat DDCS include doxorubicin, cisplatin, and/or ifosfamide. There have been several small retrospective studies offering conflicting results regarding the utility of systemic therapy in DDCS [8,9,10]. Thus, the primary objective of this study is to expand upon the current limited body of knowledge regarding the optimal systemic treatment regimen by describing treatment patterns and outcomes for patients with DDCS treated at five international sarcoma centers.

## 2. Methods

### 2.1. Study Design

This study is a retrospective review of the medical records from five sarcoma centers, including the Vanderbilt University Medical Center, Stanford University, the Washington University in St. Louis/Siteman Cancer Center, the University of Washington/Fred Hutchinson Cancer Center, and the Royal Marsden Hospital. An Institutional Review Board approved the conduct of this study. Data were abstracted from the electronic medical records of patients receiving treatment for DDCS between 2004 and 2022. 

### 2.2. Patient Population

Patients were included if they had a diagnosis of DDCS and were treated at one of the five sarcoma centers. Demographic variables, including age and sex, were collected, as well as disease characteristics, including primary disease site, primary tumor size, grade, extent of disease, treatments rendered, response to treatment, local and/or distant recurrence, site(s) of recurrent disease, and tumor mutations.

### 2.3. Statistical Analysis

Categorical and continuous variables were assessed with percentages and means; no formal statistical hypothesis testing was performed with these variables. Progression-free survival (PFS) and overall survival (OS) were estimated using the Kaplan–Meier method and were compared between groups using the logrank test. A cox proportional hazards model was used for multivariate analysis. All analyses were performed using GraphPad Prism version 6.0, R version 4, and Python version 3.0.

## 3. Results

**Figure 1:**
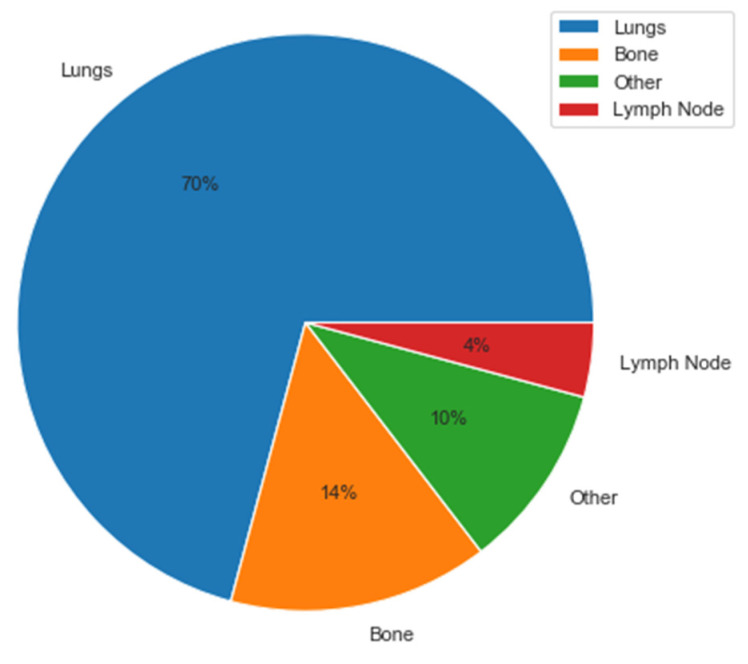
Sites of metastatic disease.

Seventy-four patients with DDCS were identified. Patient demographics and disease characteristics are outlined in Table 1. The median age of the patients was 63 years (23–87), and 61% were male. Most patients were white (n = 51; 69%). The primary tumor site was most commonly in the extremities (n = 54; 73%) followed by the abdomen or pelvis (n = 10; 14%). The median primary tumor size was 11 cm (2–34). DDCS was generally localized at diagnosis (n = 57; 77%); however, 57% of the patients who presented with localized disease (32/56) developed metastatic disease, with a median time to distant recurrence of 5.8 months. When the patients developed metastatic disease, the lung was the most common site (n = 38/42; 90%). Bone and lymph node metastases were seen in 20% and 7% of patients, respectively (Figure 1). Mutational data were not available for most patients, although IDH1/2 mutations were found in a subset of tumors. Almost all patients with localized disease underwent surgical resection (n = 56/57; 98%); however, most patients with localized disease did not receive either neo/adjuvant chemotherapy (n = 13/57; 23%) or radiotherapy (n = 15/57; 26%). Over half (n = 10/17; 59%) of the patients who presented with metastatic DDCS underwent surgical resection, and most received chemotherapy (n = 13/17; 76%). Radiation was also used infrequently in metastatic disease (29%) (Table 2). 

**Figure 2:**
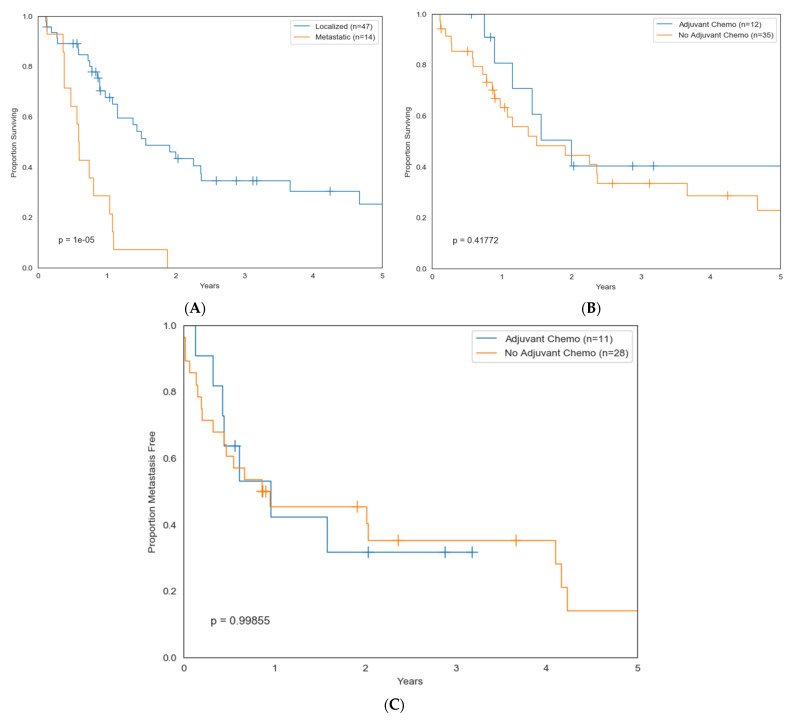
(**A**) DDCS overall survival based upon extent of disease; (**B**) DDCS overall survival based upon receipt of adjuvant chemotherapy; and (**C**) DDCS metastasis-free survival based upon receipt of adjuvant chemotherapy.

The median overall survival for patients with localized or metastatic disease was 24.8 months and 7.2 months, respectively, and the 5-year survival rate was 32% and 0%, respectively (Figure 2A). The median follow-up was 11.8 months (1.4–122). Local and distant recurrences were frequent (38% and 57%, respectively) and rapid, with a median time to recurrence of 6.6 months (1.3–114) and 5.8 months (0.7–114), respectively (Supplementary Table S1). A multivariate cox proportional hazards model (age, sex, tumor size, location, and presence of metastasis at diagnosis) identified the presence of metastasis at diagnosis as the only risk factor for worse prognosis (Table 3). Adjuvant chemotherapy (calculated for patients diagnosed with only localized DDCS) did not improve overall survival or metastasis-free survival (Figure 2B,C).

Overall, 36 patients received systemic therapy, and 13 patients received therapy in the neo/adjuvant setting, while 30 patients received therapy in the setting of metastatic disease (Table 4). Seven patients received chemotherapy in both settings. First-line regimens used for both localized and metastatic DDCS generally reflected the osteosarcoma protocols used. The most commonly used regimen was doxorubicin and cisplatin ± methotrexate (n = 15). Gemcitabine/docetaxel (n = 10) was also commonly used in the metastatic setting. The overall response rate to systemic therapy was 9% (n = 4). There were no complete responses. Four patients had a partial response. Of the responding patients, three were treated with an anthracycline-based regimen, including two patients who were treated with a combination of doxorubicin and ifosfamide, and one patient who was treated with doxorubicin and cisplatin, and one patient was treated with single-agent pembrolizumab. Most patients had progression of disease as the best response to treatment (55%, n = 26). 

There were seven patients in the study who received small-molecule inhibitors (SMIs), such as tyrosine kinase inhibitors (TKIs), mammalian target of rapamycin (mTOR) inhibitors, and isocitrate dehydrogenase (IDH) inhibitors, for the treatment of metastatic disease. Prolonged stabilization of disease (20 weeks) occurred in two patients: one who was treated with pazopanib, and one who was treated with temsirolimus. The mutational profiling of these DDCS tumors demonstrated NF2 Q410, CDKN2A/B loss, DCDKN2C L122fs*2, and EED loss in the patient treated with pazopanib, and PIK3CA E418_L422>V and TP53 R273H in the patient treated with temsirolimus.

**Figure 3:**
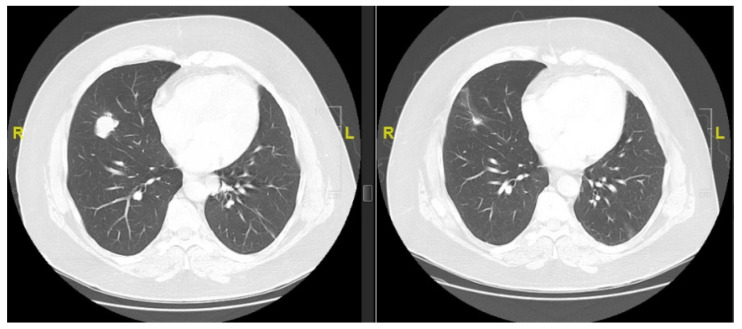
Response to pembrolizumab in a lung nodule. Scan performed after 4 months of therapy.

Five patients were treated with immune checkpoint inhibitors (ICIs). The best response was a partial response to pembrolizumab that was ongoing at the time of data cut-off (Figure 3). This patient had PD-L1 TPS of 7% and CPS of 8%, and the microsatellite status was stable. The duration of stable disease ranged from 1 to 48 weeks, including 2 patients who had prolonged stable disease (24 and 48 weeks) with ipilimumab/nivolumab and pembrolizumab, respectively. 

## 4. Discussion

DDCS is an aggressive malignancy with high recurrence rates, poor prognosis, and limited responses to currently available systemic therapy. Retrospective studies over the past four decades have consistently found a 5-year survival rate of <30% for even patients who are diagnosed initially with localized disease [5,10,11,12,13]. A recent study has shown that although tumor size is not prognostic, a percentage of dedifferentiation ≥20% and a size of dedifferentiation >3.0 cm predicted significantly worse outcomes [14]. Over this time period, there has been no significant improvement in tumor responses to treatment or in survival outcomes. Despite multiple international guidelines recommending that DDCS be treated like osteosarcoma, it is not clear that this leads to improvement in survival or in patient reported outcomes. 

In our retrospective study, we analyzed patient and tumor characteristics, as well as the treatments administered. Neo/adjuvant chemotherapy was administered to a small percentage of patients in this study but did not appear to improve outcomes for patients with localized tumors. The patients with localized disease had a very high rate of developing recurrent DDCS (75%), within an average of around six months. Other studies on localized DDCS demonstrated a similar poor prognosis. For example, in a large retrospective study of 266 patients with localized disease, the 5-year survival rate with chemotherapy was 33% (n = 76) and the rate was 25% without chemotherapy (n = 166), *p* = 0.1192. This study also found that patients with localized DDCS had a median OS of 24 months [5].

Over half of the patients in our cohort (58%) received systemic therapy for DDCS but more commonly in the metastatic setting. Three patients with a partial response were treated with a doxorubicin-based regimen, including two patients receiving a regimen in combination with ifosfamide, suggesting some activity for this regimen. Similarly, a prior retrospective study also demonstrated the efficacy of this regimen. In a review of 41 patients with DDCS, ifosfamide-based regimens were used to treat 16 patients. Based on the multivariate analysis, chemotherapy without ifosfamide was the only independent negative prognostic for disease-specific survival [12]. Similarly, the French Sarcoma Group evaluated 42 patients with DDCS and found a response rate of 20.5% to chemotherapy, with a higher response rate for combination chemotherapy (mostly anthracycline-based regimens) [8].

In our cohort, none of the ten patients treated with gemcitabine/docetaxel had a disease response. However, it is important to note that this regimen was not used as a first-line treatment. Almost all patients (90%) stopped gemcitabine/docetaxel by the end of the second cycle, suggesting limited activity of this regimen in the second-line setting. To our knowledge, the only published attempt to prospectively evaluate this regimen in chondrosarcoma was a SARC study involving patients with bone tumors [15]. This study was terminated early due to a lack of activity. 

Prolonged stable disease was seen in some patients receiving SMIs and ICIs, and a response in one patient treated with ICI. The patients who received TKIs were on treatment for a median of 11 weeks, while those treated with immunotherapy received therapy for a median of 18 weeks. These options should be investigated in a prospective manner for the treatment of DDCS, given the limited effectiveness of second-line chemotherapy. In a phase 2 study evaluating pembrolizumab in bone sarcoma, a partial response was seen in one-fifth (20%) of patients with DDCS, suggesting the possibility of activity of ICIs in DDCS [16]. Further research into the biomarkers of response for SMIs and ICIs are needed.

The limitations to our study are those that are inherent to its retrospective nature. Retrospective studies are subject to selection bias. Patients who received adjuvant chemotherapy might have had tumors with higher risk features than patients who did not receive adjuvant chemotherapy. Conversely, in the metastatic setting, patients who were less fit likely did not receive intensive therapy. Data on treatment-related toxicities and quality of life were not captured, which would be of particular importance in this cohort where intensive treatments must be balanced with the knowledge of poor prognosis. 

We believe that our study adds valuable data about patients with DDCS, filling a research gap related to different treatment regimens, especially newer options such TKIs and ICIs. Our results also suggest that doxorubicin/ifosfamide is a reasonable first-line option for DDCS and could be studied prospectively compared to doxorubicin/cisplatin. Given the poor prognosis of DDCS, we still recommend an aggressive approach with multidisciplinary evaluation. Novel treatments are desperately needed, and prospective studies are possible through multi-institutional collaboration. 

## 5. Conclusions

DDCS has poor outcomes and conventional chemotherapy has limited benefit. Future studies should focus on defining the possible role of molecularly targeted therapies and immunotherapy in the treatment of DDCS.

## Figures and Tables

**Table 1:** Patient and disease characteristics.

Patient Characteristics	All Patients (n = 74)
Median age in years (range)	63 (23–87)
Sex	
Male	45 (61%)
Female	29 (39%)
Race	
White	51 (69%)
Black	3 (4%)
Asian	2 (2.7%)
Native Hawaiian/Pacific Islander	1 (1.3%)
Unknown	17 (23%)
Sarcoma Center	
Vanderbilt University Medical Center	27
Stanford University	17
Washington University Siteman Cancer Center	12
Fred Hutchinson Cancer Center	12
Royal Marsden Hospital	6
**Disease Characteristics**	
Median tumor size in centimeters (range)	11 (2–34)
Tumor grade	
High	66 (96%)
Low	3 (4%)
Primary site	
Extremity	54 (73%)
Abdomen/pelvis	10 (13.5%)
Chest	6 (8.1%)
Head/neck	2 (2.7%)
Other	2 (2.7%)
Extent of disease at diagnosis	
Localized	57 (77%)
Metastatic	17 (23%)
Sites of metastatic disease	n = 42
Lung	38 (90%)
Bone	8 (20%)
Lymph node	3 (7%)
Other	8 (20%)
Mutation Status	
IDH1	5 (6.8%)
IDH2	2 (2.7%)
Other	2 (2.7%)
Unknown	65 (88%)

**Table 2:** Management of DDCS based on extent of disease at diagnosis.

Treatment Plan	All DDCS (n = 74)	Localized DDCS (n = 57)	Metastatic DDCS (n = 17)
Initial surgical resection			
Yes	66 (89%)	56 (98%)	10 (59%)
No	8 (11%)	1 (2%)	7 (41%)
Radiation			
Yes	20 (27%)	15 (26%)	5 (29%)
No/unknown	54 (73%)	42 (74%)	12 (71%)
Chemotherapy			
Yes	26 (35%)	13 (23%)	13 (76%)
No/unknown	48 (65%)	44 (77%)	4 (24%)

**Table 3:** Association between patient characteristics at diagnosis and prognosis.

Variable	Hazard Ratio	95% CI	*p*-Value
Age > 60 years	1.2	0.68–2.1	0.53
Sex (Male)	1.35	0.76–2.4	0.3
Tumor Size (cm)	1.01	0.95–1.07	0.74
Location (Extremity)	1.37	0.7–2.7	0.36
**Metastasis at Dx**	**4.7**	**2.3–9.3**	**0.00001**

**Table 4:** Systemic regimens used by timing.

Systemic Treatment Regimen	Number of Patients	Median Weeks of Treatment (Range)	Best Response to Systemic Therapy
Neo/adjuvant *	13		
Doxorubicin/cisplatin	6	10.5 (3–16)	SD
Doxorubicin	4	11.5 (6–18)	NA
Methotrexate/doxorubicin/cisplatin	3	8 (4–16)	SD
Doxorubicin/methotrexate	1	8	NA
Doxorubicin/carboplatin	1	20	NA
Doxorubicin/ifosfamide	1	18	NA
Gemcitabine/docetaxel	1	18	NA
Imatinib	1	2	NR
Metastatic *	30		
** *Chemotherapy***			
Doxorubicin/cisplatin	12	7.5 (2–23)	PR
Gemcitabine/docetaxel	10	6 (0.5–22)	SD
Doxorubicin	4	10.6 (6–18)	SD
Doxorubicin/ifosfamide	3	9 (9–18)	PR
Methotrexate/doxorubicin/cisplatin	3	9 (8–10)	PD
Ifosfamide/etoposide	2	6	PD
Doxorubicin/olaratumab	1	19	SD
Doxorubicin/carboplatin	1	12	PD
Gemcitabine	1	1	PD
Ifosfamide	1	15	PD
** *Small-molecule inhibitors (SMI)***			
Pazopanib/regorafenib/sorafenib	6	11 (6–20)	SD
Everolimus/temsirolimus	2	16 (12–20)	SD
IDH inhibitor	1	NR	NR
** *Immune checkpoint inhibitors (ICI)***			
Ipilimumab/nivolumab	4	11 (1–24)	SD
Pembrolizumab	5	21 (12–48)	PR
Atezolizumab	1	12	PD
** *SMI + ICI***			
Pazopanib + pembrolizumab	1	8	PD

* Patients received multiple regimens.

## Data Availability

Data will not be made publicly available.

## Supplementary Materials

The following are available online at https://www.mdpi.com/article/10.3390/cancers15092617/s1. Table S1: Recurrence rates for patients who presented with localized DDCS.
