# Supplementary material for: A Retrospective Multi-Institutional Cohort Analysis of Clinical Characteristics and Outcomes in Dedifferentiated Chondrosarcoma
Source: Cancers (Basel). 2023 May 5;15(9):2617. doi: 10.3390/cancers15092617 (PMC10177459; doi:10.3390/cancers15092617)
Supplement: Supplementary file 1 [file cancers-15-02617-s001.zip › cancers-2296687-supplementary.pdf]

cancers MDPI

Table S1. Recurrence rates for patients who presented with localized DDCS.

Recurrence type	Localized DDCS N=57	Median time to recurrence in months (range)
Local		
Yes	21 (38%)	6.6 (1.3-114)
No	36 (62%)	NA
Distant		
Yes	32 (57%)	5.8 (0.7-114)
No	25 (43%)	NA

*Patients received multiple regimens.
